# Elements of Descriptive and Practical Anatomy

**Published:** 1829-01-01

**Authors:** 


### ELEMENTS OF DESCRIPTIVE AND PRACTICAL ANATOMY, FOR THE USE OF STUDENTS. By *Jones Quain*, A.B. M.B. Member of the Royal College of Surgeons, &c. &c. 8vo. pp. 700. 1828.

IN this work, which has already attracted considerable notice by its merits, it has been the aim of the author, to give a condensed and methodical description of the different structures and organs which enter into the composition of the human body,—to point out the most convenient methods of conducting their anatomical investigation—to indicate some of the more important practical applications that may be made of the facts disclosed to the student during the progress of his inquiries—and to present abridged summaries of the most instructive principles of general anatomy.—After some excellent observations on the objects of anatomy, the proper mode of studying it, and its importance to the practitioner, together with a short history of the growth of organized bodies, and their elementary textures; the author proceeds with the anatomy, general and descriptive, of the osseous and ligamentous systems. He then considers the anatomy of different parts of the body, commencing with that of the parietes of the abdomen, and lower extremity; of which he first describes the muscles, then, in succession, the arteries, veins, lymphatics, nerves, and membranes; giving directions for the procedure of the student in their dissection, and descriptions of the operations the surgeon may be called upon to perform in these regions. The parts next similarly reviewed, are the back, the upper extremity, the head and neck, the abdominal viscera, and peritoneum, the urinary organs, the organs of generation, the perineum, the thorax and contained viscera, the brain, cerebral and sympathetic nerves, and the organs of the senses:—an arrangement we much approve of, as in this, unlike most other authorities which the student carries with him into the dissecting-room, he will not be obliged to seek in different chapters for the description of parts which his knife discloses to him all grouped together, and as by studying them in connexion, the knowledge of one fact is brought to support, or renew the recollection of another. Of the advantages of this mode of instruction, our author seems fully sensible, and he has lost no opportunity of turning it to account. Even in alluding to incisions through the integuments, many important points of information are brought to the recollection of the student.

Some of the systems of anatomy most generally read, are so arranged as if a knowledge of the anatomical characters of bones, muscles, arteries, &c. constituted the ultimate object which we seek to attain; thus, adding to the acknowledged dryness of some parts of the study, without any endeavour, on the part of the author, to impress on the memory a remembrance of the facts conveyed. In the work before us, the descriptive part is so connected with its practical application as to give an increased interest to both, and embodied in language so simple and perspicuous, as to bring the reader easily and pleasantly acquainted with the subjects under consideration. However, to give some idea of the author's style, we shall make a few extracts, commencing with his description of the formation of the teeth.

"In early infancy, the margin of each jaw forms a continuous ridge, covered by the mucous membrane of the mouth; but when examined internally, it is found to contain a number of membranous follicles, separated by their partitions. Each of these is composed of two membranes; the internal one, thin and firm, forms a shut sac, and receives the ramifications of the dental vessels; the external one, thick and spongy, appears to be continuous with the periosteum of the jaw at the border of the socket, into which it descends, lining it as far as the point where the vessels enter; on these it is reflected, and ascends to the internal sacculus, the exterior of which it invests as far as the entrance of the vessels; where it is again reflected to the alveolus, which it lines. Such is the general conformation of the sacculi up to the fourth month of fœtal life; at which period the process of ossification begins, and proceeds as follows. At the bottom of the sacculus (adopting that term as a means of distinguishing the internal membrane, or shut sac, from the external one which is reflected over it) a soft vascular mass arises, supplied with vessels and nerves, derived from those ramified on the sacculus. This mass gradually increases, and assumes the form of the tooth, and constitutes, as it were, the model on which it is to be moulded. The osseous matter begins to be deposited, first on its free surface, in a thin lamella, corresponding with the crown of the tooth; or rather, in dots or scales, corresponding with each of the inequalities on its upper surface, and gradually uniting to form a lamella. When the breadth is completed, the growth in length begins, and extends from the crowns to the roots, by successive depositions on the exterior of the pulp; so that it may be said to clothe itself in osseous matter, the roots being the parts last formed, the crown first. The bony lamella is at first remarkably thin, and the contained cavity of considerable size; but, as the former increases in thickness by successive depositions on its inner surface, the latter and its inclosed pulp, become proportionally diminished, until the roots and body of the tooth have acquired their proper degree of thickness. Thus the principle of eccentric development obtains throughout the entire process of the growth of the teeth, the particles of matter being first deposited at the points most remote from the centre: still, it is not strictly analogous with what builders term over-hand work, for it not only proceeds from without to within, but begins at the top, and thence descends to the bottom. This principle should never be lost sight of by those who enter on a consideration of the theories of life and organization."

Here the author has neglected to mention, for we presume he must be aware of the fact, that when the rudiments of the temporary teeth are somewhat advanced, new sacculi are sent off in succession, for the formation of the permanent teeth, at the under and inner part of the sacculi of the temporary teeth of the upper jaw, and at the superior and inner part of those of the lower jaw, the new sacculi lying between those of the temporary teeth and the internal alveolar plate, each being on the inner side of the tooth it is to succeed, and connected with the gum.

After having given a masterly and concise summary of the history of the operation of lithotomy, of the modes adopted by Celsus, Johannes Romanus, Frere Jaques, Raw, and Cheselden, Mr. Quain thus describes the operation.

"The operation, as devised by this eminent surgeon, (Cheselden,) and practised at the present day, may be performed as follows, (attention is here confined to the operative part exclusively, all other details and preliminaries being foreign to our present purpose):—A grooved staff, corresponding in size with that of the urethra, is in the first place introduced, and so placed, that its concavity lies close beneath the pubic arch, whilst its convexity is turned somewhat towards the left side of the perineum. The edge of the scalpel is then laid on the skin, close by the left side of the raphé, at about fourteen lines before the margin of the anus, and is thence drawn downwards and outwards to the interval between the margin of the anus and the left tuber ischii, inclining a little nearer to the latter: this divides the skin and superficial fascia. The second incision, commencing a quarter of an inch, or a little more, according to the size of the bulb, below the upper end of the first, is carried downwards in the same direction and extent, and so divides the lower fibres of the accelerator muscle, the transversalis perinei muscle and artery, with part of the levator ani and deep perineal fascia. The staff should now be sought for at the upper angle of the incision: if the bulb be large, it will be necessary to press it aside with the index finger of the left hand. The point of the scalpel being elevated, by depressing its handle, and throwing the hand a little backward, it is made to enter the groove of the staff, its lodgment therein being ensured by moving it slightly from side to side, and then is passed along the groove, so as to lay open the membranous part of the urethra. When this has been effected, the operator draws downwards to himself the handle of the staff, (its concavity being held securely beneath the pubic arch,) by which means its beak is made to move upwards and backwards, and so removed from the rectum. Whilst this is being done, the scalpel (previously lateralized, so that the direction of its edge shall correspond with that of the external incision) is made to slide along the groove, dividing in its passage the prostatic part of the urethra and neck of the bladder. After this has been completed, the scalpel is withdrawn a little, and carried obliquely downwards in the direction of the first incision, so as to divide any septa that may lie across the wound, which usually consist of part of the fibres of the levator ani, and of the transversus perinei, if not severed in the second incision. The advantage, or rather the necessity, of freely dividing the membranous part of the urethra, previously to depressing the staff, has been put on a very clear point of view by Mr. Colles:*—If you have entered your knife into the urethra high up in the perineum, and while the point of the knife is lodged there, should depress the staff, and attempt the division of the prostate, you will have to make it describe the portion of a circle at the same time that it is dividing very resisting parts."

The author next adverts to the mode of proceeding recently suggested, of reducing by mechanical trituration (with the lithontripteur) the stone into particles so small as to admit of their being expelled with the urine. After describing the instrument and its modus operandi, he discountenances its use, as a hazardous and doubtful measure, reasoning thus:—

* Treatise on Surgical Anatomy, p. 211.

"Now, suppose every thing to be done in the way of trituration and bruising that an instrument can do, we may just raise this question. What are the immediate effects of the operation, and what are its remote consequences? The urethra and neck of the bladder, both sensitive in no small degree, having suffered during the operation a degree of irritation proportioned to the susceptibility of the individual, and of the part, from its previous condition, begin now to be subjected to the additional irritation consequent on the passage, from day to day, for an indefinite period of time, of a quantity of sabulous and gritty matter over their surface. After all this, should a fragment of any size remain, (an occurrence far from unlikely,) the local and constitutional irritation may be such, that the triturating process cannot, or will not, again be submitted to, and then the operation by incision must be resorted to, under most unfavourable circumstances. If the calculus be large, or of the mulberry species, it would require a considerable force to crush it, even though it were perforated in several directions; and if, in the endeavour to accomplish this, one of the prongs were to give way, (an accident not unlikely to happen to those not habituated to the use of such instruments,) the operation by incision cannot be deferred, even though it becomes necessary to lay open the bladder irritated by the previous proceedings. To conclude, this expedient cannot, under any view of it, be considered as a substitute in all cases, nor even in the majority of cases, for the operation by incision. It cannot be resorted to in persons of an irritable habit, or in cases in which the bladder has become contracted and irritable by the long continuance of a calculus within it; neither is it practicable in children, for in them the urethra will not admit, without great suffering, a straight instrument, nor will it carry one of sufficient size to admit of its prong being stout enough to exert any considerable force on the stone. It is applicable, then, only in cases that may be considered free from local or constitutional irritation, and even in such, it may induce both without ensuring a complete removal of the stone."*

We quote the following passage, as being well calculated to shew the philosophic turn of mind with which our author generally approaches his subject. After describing the concentric lamellæ of the cornea, and its interstitial fluid; this structure, he observes, "is beautifully adapted to the functions of the eye. Were it fibrous, like the sclerotica, even though, at the same time, translucent, it would cause a dispersion of the rays of light, and thereby resolve them into their primitive or prismatic rays, which would necessarily produce a coloured and confused image. The effect may readily be exemplified by looking at a lighted taper through a feather; the flame will instantly appear surrounded by a halo of coloured images; and were the cornea made up of fibres woven ever so finely, the effect would be similar."

We shall close this short notice of the work with the following observations on the pulse.

"The flow of blood in the arteries determines a peculiar phenomenon called *pulse*. The number of arterial pulsations in a given time varies, according to the age, sex, and constitution of each individual; but, setting aside these and all other variations, dependent on different circumstances, in health as well as in disease, when we come to inquire what is the change sustained by the arteries during their pulsation, and what is the cause of that change, we find that, even on these preliminary questions, a considerable difference of opinion exists among physiologists. According to Mr. Hunter,† 'arteries, during their diastole, increase much more in length than in width. It is, however, the increased diameter that is perceived by the touch.' According to Dr. Hales,‡ 'at each systole of the heart, the blood in the arteries is propelled forwards with an increased impetus thereby dilating the canal.' Dr. Thomson's§ opinion is nearly to the same effect. 'The pulsation of

* Reasoning must give way to facts. The operation is every day performed on the Continent with complete success, and with very little pain, by M. Civiale and others. Sir Astley Cooper's testimony, from ocular demonstration, will be seen at page 192 of this number of our Journal.—*Editor.*

† On the Blood and Inflammation, p. 23.

‡ Hæmastatics.

§ Lecture on Inflammation, p. 63.

arteries is derived entirely from the dilatation and elongation which they experience from the blood impelled into them by the systole of the heart.' Haller, in one of his Elements, attributes the pulse to a dilatation of the arteries, but, subsequently, admits that the dilatation cannot be perceived, and is only inferred from the sensation communicated to the finger. Bichât, during the progress of his researches, frequently examined the condition of denuded arteries; and, as he could not observe any adequate dilatation in them, he came ultimately to the conclusion, that the pulse was attributable to a locomotion in the whole artery, ('un mouvement de totalité,'*) which causes it to spring against the finger during the systole of the heart. Yet he still inclines to admit a dilatation in some degree;—'Quant à la dilatation, elle est *presque* nulle dans l'etât ordinaire.' It is scarcely necessary to allude to the hypothesis of Dumas,† that arteries are endued with a power of active dilatation, which enables them to expand, to receive the blood which is about to be propelled into them. The late Dr. Parry,‡ of Bath, assisted by Mr. Norman, made a great number of experiments, to determine the condition of arteries during their pulsation. Whilst prosecuting his researches, he laid bare fifty-five of the larger arteries, in horses, sheep, and dogs, 'yet could not, by any mode of scrutiny, detect, in a single instance, the smallest dilatation or contraction, corresponding with the systole or diastole of the left ventricle of the heart.' It is more than probable, as Dr. Wilson Philip has remarked, that the mode of measurement adopted in these experiments was not sufficiently delicate to ascertain the point with precision, for Dr. Hastings found that a ligature, placed round an artery, was tightened at each contraction of the heart. It appears, then, that the weight of testimony and authority inclines to that opinion, which attributes the pulse to a dilatation and elongation of the artery, synchronous with the systole of the ventricles. It cannot be said to be actually dependent on that cause, for, if it were, no difference could exist in the state of the pulse in the corresponding arteries in different limbs, neither could it be raised above par, as we frequently find it, in a vessel leading to an inflamed part, nor depressed below it in a paralytic limb."§

Mr. Quain, in this, as in other doubtful points, states the facts and theories that different authorities have brought forward, making such comments on them as will lead the reader to the most rational conclusion. He has freely availed himself of the writings of the Continental authors, as well as of those of our own country, and enriched his work with their opinions and discoveries, making it an abridged summary of all the established facts and admitted principles in the present state of anatomical science. Though written designedly for students, we can conscientiously recommend it to practitioners, as an excellent book of reference; and, to pupils, as a most useful assistant in their operations in the dissecting-room, and a no less instructive companion in the studies of their chamber.

---

* Anatomie Générale, vol. ii. p. 335.

† Physiologie, vol. iii. p. 314.

‡ Essay on the Pulse.

§ Long and attentive consideration of this subject has led us to conclude, that the sensation communicated to the finger, called the pulse, is produced simply by the rush of blood along the artery at each ventricular contraction. If the hand or a finger be applied to a hose, leading from an engine or pump, the sensation of a pulse, in the most perfect degree, is communicated at each stroke of the piston, though the hose remains full in the intervals.—Ed.

